# Strong Associations Between Childhood Victimization and Community Violence in Male Forensic Mental Health Patients

**DOI:** 10.3389/fpsyt.2020.628734

**Published:** 2021-02-01

**Authors:** Roar Fosse, Gunnar Eidhammer, Lars Erik Selmer, Maria Knutzen, Stål Bjørkly

**Affiliations:** ^1^Division of Mental Health and Addiction, Vestre Viken Hospital Trust, Drammen, Norway; ^2^Center for Research and Education in Forensic Psychiatry, Oslo University Hospital, Oslo, Norway; ^3^Faculty of Health and Social Sciences, Molde University College, Molde, Norway

**Keywords:** childhood adversities, polyvictimization, maltreatment abuse chronology of exposure scale, interpersonal violence, forensic mental health

## Abstract

**Background:** Childhood abuse and neglect increase the risk of both mental disorders and violent behavior. Associations between child relational adversities and violent behavior have not been extensively investigated in forensic mental health settings. We asked whether the extent of child adversities predicts the extent of violence in the community in forensic mental health patients.

**Methods:** We included 52 male patients at a medium security forensic mental health ward, with diagnoses of predominantly paranoid schizophrenia and other schizophrenia and psychotic disorders. Seventy-five percent had comorbid substance abuse. We extracted information on six types of child adversities based on clinicians' administrations of the Historical Clinical Risk Management 20 version 3 (HCR 20) scale and summary notes in electronic patient journals. These same sources were used to extract information on war trauma and interpersonal violence in the community. We established cumulative scales for exposure to number of types of child adversities and number of incidents of community violence.

**Results:** Physical and emotional abuse, emotional and physical neglect, and bullying were associated with higher levels of community violence. We observed a linear, significant increase in the frequency of community violence with cumulative numbers of child adversity types.

**Conclusions:** Cumulative exposure to child adversities may be associated with higher degrees of violence in forensic mental health patients, with the most violent patients having the most extensive exposures to adversities. An enhanced focus on child adversities in risk assessment and management of violence may be considered in forensic inpatient settings.

## Introduction

Violent behavior combined with severe mental health problems complicate the treatment of many forensic mental health patients. In forensic settings of compulsory treatment, often following court mandates, central clinical challenges include establishing therapeutic relationships and agreements about working toward common goals of managing aggression and violence and key risk factors such as substance abuse (1–3). Much is to gain for both patients, treatment services, and society by improved understanding of developmental pathways of these patients' problem complexes.

Risk factors for violence with high predictive validity in forensic mental health patients include psychotic symptoms, substance abuse, criminal behavior, and prior violent behavior (4, 5). In the last decade, increasing evidence has pointed toward an important role also for childhood abuse and neglect. In the general population, being victim of child maltreatment increases the risk of violent and criminal behavior later in life (6, 7), an intergenerational transmission pattern referred to as the “cycle of violence” (8–10). Child victimization also increases the probability of the entire array of mental health problems, including personality disorders, psychosis, and schizophrenia as well as substance abuse that are prevalent among forensic mental health inpatients (11–14). In people with severe mental disorders, those exposed to the most extensive child maltreatment have an increased propensity of later being violent toward other people, mirroring the association found in the general population (5, 15–18).

High rates of various types of child victimization are reported also in forensic mental health patients, with maltreatment reported for 75–85% (19–23). A handful of studies have addressed the pertinent question of associations between child victimization and violent and criminal behavior in forensic patients. To the best of our knowledge, there exist only two publications addressing associations between victimization and violent behavior executed in the community in this group. Dudeck et al. (24) studied long-term forensic inpatients with diagnosis predominantly of substance abuse and personality disorders. They found no associations between childhood maltreatment and the prevalence of homicide, robbery, and grievous bodily harm. Bruce and Laporte (25), in contrast, reported that in forensic inpatients with diagnosis mainly of schizophrenia and schizoaffective disorder, those who had been victimized as children compared to those who had not had a 170% increased risk of having been violent in the community prior to hospital admission. Three other studies assessed the association of child victimization with patient violent behavior at forensic mental health wards during hospital stays. Hoptman et al. (26) studied patients with various diagnosis including schizophrenia (58%) and substance abuse (57%), and reported that those who were the most violent on the ward more often than other patients had been physically abused during upbringing. Hammer et al. (27) found that long-term forensic patients with the highest level of aggression and violence on the ward more often had been sexually and physically abused during childhood than less aggressive and violent patients. Macinnes et al. (20), however, observed no significant association between child maltreatment and verbal and physical aggression during hospital stays in patients with mainly schizophrenia diagnosis.

Several factors may account for the variable findings in the small set of previous studies of associations between victimization and violence in forensic mental health patients. The studies targeted different types of violence and criminal behavior. They studied forensic patient groups with different types and degrees of mental health problems, measured childhood victimization in different ways, and some studies may have included selective patient subgroups because only some patients provided informed consent to research. We suggest that two methodological features may be particularly important to derive at a more consistent picture. First, different types of childhood maltreatment tend to co-occur, and polyvictimization scales may better capture associations with outcome variables than dichotomous measures of single child adversity types (28–31). Second, compared to dichotomous present–absent scales for violence, graded scales that measure different degrees of violence may better capture associations with child adversities (27).

By incorporating the above noted method components, we aimed to investigate associations between childhood victimization and community violence prior to hospital admission in forensic patients. We asked whether, when analyzed within the patient group, (i) the presence of single types of child relational adversities was associated with more frequent community violence, and (ii) whether a graded association existed between the cumulative number of child adversities that patients were exposed to (polyvictimization) and the frequency of community violence.

## Materials and Methods

We report from a cross-sectional, retrospective study exploring within-group associations between childhood victimization and violent behavior prior to admission in forensic mental health inpatients. Data were extracted from an observational quality assurance registry at Blakstad hospital, Vestre Viken health trust, encompassing all patients admitted to the forensic psychiatric unit between June 1, 2016 and December 31,^.^2019. Data were based on registrations in electronic patient journals (EPJs). The setting was a 12-bed medium secured forensic mental health ward, serving a catchment area of about 500,000 inhabitants in a mostly rural and suburban region that includes five small cities (15,000–100,000 inhabitants). The forensic unit admits patients with a combination of severe mental illness and definite or assumed violence risk, as referred from other psychiatric wards at the Vestre Viken Hospital region, community mental health services, prison services, or following court order of conviction to treatment.

### Ethics

Data for this study were extracted from a quality register at the forensic mental health ward approved by the hospital Data Protection Office for research (ref. nos. 16/00117-13 and 16/00117-146). The register was established to assess early signs of violence in the admitted patients, evaluate use of the Early Recognition Method to prevent violence (32), and develop and evaluate individual ways to reduce violent behavior. The Data Protection Office approved publication of the current results from the register (ref. no. 20/09213-1).

### Participants

Altogether 57 patients were admitted to the forensic ward during the study period. Of these, 52 were male, and five were female. Due to the low number of females and considerable differences between males and females in violent behavior, we included only male patients in this study. Table 1 provides the characteristics of the 52 male participants. The most common primary diagnoses (ICD-10) as set by trained clinicians at the ward were paranoid schizophrenia (F20.0, *n* = 27), other schizophrenia diagnoses (F20.1–20.9, *n* = 4), and diagnosis of other psychotic disorders (F21–29, *n* = 9). Eight patients were given a secondary diagnosis of personality disorder (F60–69), five of these were antisocial personality disorders (F60.2). Based on clinical assessments that included the Alcohol Use Disorders Identification Test (AUDIT) and Drug Use Disorder Identification Test (DUDIT) (33, 34), 39 patients were identified with substance abuse problems (Table 1).

**Table 1:** Characteristics of 52 male forensic inpatients.

**Participant characteristics**	
Age (mean, SD)	35.7 (8.8)
**ICD-10 diagnosis (*****n*****, %)**	
Paranoid schizophrenia (F20.0)	27 (51.9%)
Other schizophrenia diagnosis (F20.1–20.9)	4 (7.7%)
Other psychotic disorders (F21–29)	9 (17.3%)
Diagnosis in other domains: F0–19, F31, or F70–90	9 (17.3%)
No diagnosis due to short stays	3 (5.8%)
Comorbid personality disorder (F60.2, F60.3, F61)	8 (15.4%)
Substance abuse (*n*, %)	39 (75%)
Months at the ward during the 3.5-year study period (mean, SD)	10.6 (12.7)
**Ethnicity (*****n*****, %)**	
Norwegian	29 (55.8%)
Other European	5 (9.6%)
African	8 (15.4%)
East Asian	10 (19.2%)

### Variables and Measures

#### Abuse and Neglect During Upbringing

We based our scoring of childhood relational adversities on a two-step process. In the first step, we identified child adversities as reported in the EPJs. Here, the primary source was version 3 of the Historical Clinical Risk Management 20 scale (HCR 20) (4). The HCR 20 is a structured professional judgment tool to assess and manage violence risk (35) and was completed by each patients' treating clinician, a psychologist or psychiatrist. Item 8 in the HCR 20 addresses traumatic experiences, separated into two subcategories: (a) Victimization/trauma and (b) poor parenting/caregiving. According to the HCR 20 instructions, the victimization/trauma part includes the experience during upbringing (up to age 18) of the following: Sexual, psychological/emotional, or physical child abuse or neglect committed by parents, other primary caregivers or other adults; violent victimization committed by any person; peer harassment/bullying; and any other interpersonal violence and victimization including military experiences. Scorers are instructed to pay particular attention to the severity (persistency, repetitiveness, or chronicity) of the experiences. The poor parenting/caregiving subcategory includes the experience during upbringing of coercive or overly harsh parental discipline; witness to frequent, severe parental conflicts or to violence against loved ones (siblings, other family members, and close friends); parental substance abuse; parental criminality and convictions; unstable households, foster home placements, and institutional raising. The HCR 20 item 8 as well as its two subcategories have good to excellent interrater reliability when scored by experienced clinicians at forensic mental health wards (4). In addition to the HCR 20, we searched for further information on child adversities depicted in summary notes (epicrisis) in EPJs written by specialists in psychology at the forensic ward or at other wards or outpatient units where the patients previously had been treated.

In the second step, we sorted the information about child adversities from the HCR 20 and EPJs into different exposure types, starting out with 10 types defined in the Maltreatment and Abuse Chronology of Exposure (MACE) scale (36–38). The MACE is a well-validated scale that assesses a wider extent than most other existing scales of exposure to child adversities before age 18. We merged some of the 10 MACE types (e.g., emotional harassment by peers and physical harassment by peers) into single types, resulting in the six types described in Table 2.

**Table 2:** Scoring of six types of relational adversities in upbringing based on the MACE scale.

**Child adversity type**	**Description/examples^***a***^**
Emotional abuse by parents/other caregivers	Repeatedly swore, yelled, or screamed at person, called person names, threatened, said insulting or hurtful things, kept important secrets or facts from person, locked person in a closet, attic, basement, garage, etc.
Physical abuse by parents/other caregivers	Repeatedly hit person with hands or objects, kicked, severely spanked, shoved, slapped, etc.
Emotional or physical neglect/care failure—by parents/other caregivers	Parents/care givers were emotionally unavailable, did not have the time or interest to talk to person, did not give one the feeling of being loved/important, was not protected/cared for/looked after/supported, did not have enough to eat, had to wear dirty clothes, was left to oneself
Sexual abuse by caregivers or other adults	Sexually touched/fondled, had to sexually touch an adult, adults attempted or actually had sexual intercourse with person (oral, anal, and vaginal)
Witness to violence against others	Witnessed parents, stepparents or other adults living in the house (repeatedly) doing hurtful things to one's mother, father, other care givers, siblings etc.: intentionally harmed, pushed, grabbed, slapped, pinched, hit, kicked, threatened to harm, severely quarreled with them
Bullying verbally or physically by peers	Children/adolescents of the same age did the following to the person: repeatedly swore at, called names or insulted, said hurtful things, humiliated, spread rumors or posted derogatory messages, intentionally excluded from activities or groups, threatened to physically hurt or take ones possessions, forced one to do things, intentionally hit, kicked, pushed, grabbed, slapped person, forced person to sexual activity

a *The descriptions and examples are derived from wordings of single items in the Maltreatment and Abuse Chronology of Exposure (MACE) scale*.

In scoring child adversities, two raters (GE and LES) first extracted relevant information from HCR 20 item 8 and summary notes in the EPJs, writing down all available details of victimization. They then separately scored one-half of the patients for the presence vs. absence of each of six maltreatment types derived from the MACE scale, before meeting to decide upon consensus scores for each of these six types. For statistical analysis, we calculated a total score for the number of maltreatment types experienced by each participant (polyvictimization), ranging from 0 to 6.

#### War Trauma

We extracted information also on war trauma from EPJs. We included reports, in war settings, of persecution, kidnapping and being held as hostage, torture, directly threatened with weapons, physically injured, and having witnessed violent war situations. War trauma was scored by the same two raters who scored MACE type victimization, using the same procedure.

#### Violence

We defined acts of violence toward people according to the HCR 20, as actual attempts or threats of physical or severe psychological damage toward another person, done on purpose and being unwanted/unauthorized. We used a 0–4 scale for the number of violent acts toward people in the community prior to admission to the forensic ward. A score of 0 was given when no violent acts were identified, a score of 1 when 1–2 such acts were identified, a score of 2 represented 3–10 identified violent acts, 3 represented 11–20 such acts, and 4 represented 21 or more acts.

Two of the authors (GE and LES) each scored half the patients on number of interpersonal violent acts (0–4 scale) using information in EPJ's and HCR 20. For 20 of the patients, we tested interrater reliability for the violent acts scale, which yielded a 100% agreement.

### Statistical Analysis

Very few patients had <3 violent acts on the scale for interpersonal violence. We therefore collapsed the first two score levels on this scale (0 for no violence and 1 for 1–2 violent acts) into the score of 1, leaving a four-point 1–4 scale that we used as outcome measure in all statistical tests.

We first used independent sample *t*-tests to investigate associations with violent acts for each of six types of child adversities and for war trauma. Next, we carried out a multiple linear regression analysis with the maltreatment scale (0–6), war trauma (yes, no), substance abuse (yes, no), diagnosis (paranoid schizophrenia—*n* = 27, other diagnosis—*n* = 25), and age as predictors for violent acts (1–4 scale). All variables were tested together. We checked for multicolinearity using the variance inflation factor (VIF) and inspected QQ plots to assess distribution of residuals for the violent acts scale.

## Results

Table 3 summarizes the prevalence of child adversities and war trauma, and incidents of community violence. We identified at least one of the six child adversity types in 32 of 52 patients (61.5%), with a mean number of 2.1 types (median 1.0, SD = 2.1) per patient. While war trauma was found for 11 patients (21.2%), 36 patients (69.2%) were identified with either child adversities or war trauma. We found at least one violent attack on other people in the community prior to admission in 50 out of the 52 patients (96.2%; Table 3).

**Table 3:** Prevalence of child adversity, war trauma, and violence.

**Variable**	***N* (%)**
Any child adversity	32 (61.5)
Bullying	29 (55.8)
Emotional neglect	21 (40.4)
Emotional abuse	15 (28.8)
Physical abuse	14 (26.9)
Sexual abuse	2 (3.8)
Witness to violence	17 (32.7)
War trauma	11 (21.2)
Any incident of violence	50 (96.2)
No incidents	2 (3.8)
1–2 incidents	5 (9.6)
3–10 incidents	20 (38.5)
11–20 incidents	9 (17.3)
21 or more incidents	16 (30.8)

In *t*-tests, exposure to several of the child maltreatment types were associated with more incidents of community violence. This was seen for physical abuse, *t*_(50)_ = 3.13, *p* = 0.003, emotional abuse, *t*_(50)_ = 2.82, *p* = 0.007, emotional/physical neglect, *t*_(50)_ = 2.92, *p* = 0.005, and bullying, *t*_(50)_ = 2.19, *p* = 0.034. In contrast, war trauma was associated with less violence at a statistical trend level, *t*_(50)_ = −1.69, *p* = 0.098.

**Figure 1:**
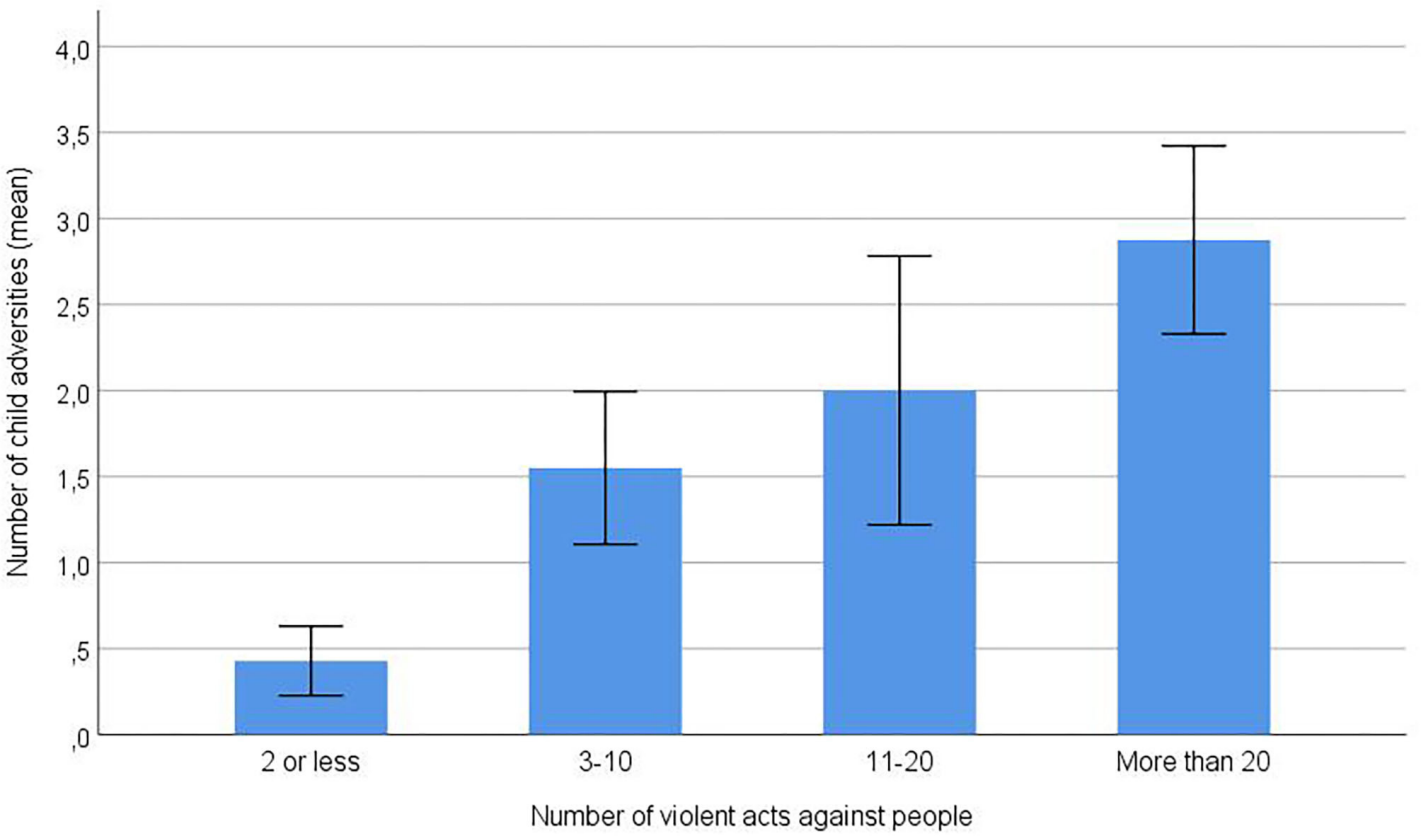
Number of child adversity types (0–6 polyvictimization scale) in subgroups of 52 forensic mental health patients with varying numbers of violent community acts. The most violent patients (score of 4 on the cumulative violence scale, *n* = 16) had 6.7 times higher mean score on the polyvictimization scale (mean = 2.88) compared to the seven least violent patients (score of 1 on the cumulative violence scale, mean polyvictimization score = 0.43). Error bars: ±1 standard error of the mean.

In the linear regression analysis, multicolinearity was not present among the predictors, with VIF values between 1.02 and 1.44. Inspection of a QQ-plot showed that residuals for the violent acts scale were approximately normally distributed. We identified significant contributions upon incidents of violence for the polyvictimization scale (0–6), with a one standard deviation increase on this scale corresponding to a 0.44 standard deviation increase on the violence scale (Table 4; see Figure 1 for an illustration). In contrast, the presence of war trauma was associated with less violence at a statistical trend level.

**Table 4:** Associations of cumulative childhood maltreatment (0–6 polyvictimization scale) and war trauma with violent behavior in 52 forensic mental health inpatients.

	**Beta**	**95% CI**	***p***	**β**
Polyvictimization scale	0.22	0.08 0.37	0.003	0.44
War trauma	−0.65	−0.1.33 −0.02	0.059	−0.25
Substance abuse	−0.21	−1.00 0.59	0.604	−0.09
Diagnosis	−0.14	−0.70 0.41	0.609	−0.07
Age	0.01	−0.03 0.05	0.582	0.08
Adjusted *R* squared	0.15			
*F*_5, 46_ = 2.74, *p* = 0.030				

## Discussion

We studied the impact of child relational adversities on community violence in 52 male forensic inpatients. The majority of patients were diagnosed with psychotic disorders and had co-occurring substance abuse, which are significant, established risk factors for violent behavior. In this high-risk group, the frequency of community violence was higher when patients had been exposed to each of child physical abuse, emotional abuse, emotional and physical neglect, and bullying. Moreover, the frequency of community violence increased linearly with accumulated exposure to multiple types of child adversities.

Our findings for community violence are in line and extend those of Bruce and Laporte (25) who reported that child victimization was more prevalent in forensic patients who had been violent in the community prior to hospital admission, compared to those who had not. Also consistent with our results are two out of three studies of forensic patients that reported associations between child victimization and violence at the ward during hospital stays (20, 26, 27). Among these studies, Hammer et al. reported that not only the presence but also the severity of violence was associated with stepwise increased extent of child victimization, mirroring our finding of graded associations when taking into account the frequency of violent acts.

We did not find that war trauma was associated with community violence. On the contrary, in regression analysis with control for concomitant effects of cumulative child victimization, we observed a trend for war trauma to be associated with less community violence. We are not aware of other studies on how war trauma affects violence risk in forensic patients, with more studies needed to illuminate this issue.

### Clinical Understanding and Implications

The associations that we observed between relational adversities during upbringing and violent perpetration suggests that sequels of childhood (poly) victimization are relevant to the clinical understanding of violent forensic mental health patients. These trauma sequels may include disturbed attachment patterns, low self-esteem, psychological distress and increased stress sensitivity, suspiciousness to other people, hypervigilance to potential threats, attention bias toward or away from threats (avoidance), dissociation, PTSD symptoms, impaired learning and verbal abilities, emotional dysregulation, and reduced self-control (29, 39–41). Hiday (42) suggested that such trauma sequels may interact with social, neurobiological, and life-style factors and with mental health disorder strain such as threat/control override symptoms (43). In Hiday's (42) model, maltreated people, for example those who have been bullied, emotionally abused, and physically abused, may be more prone to appraise situations antagonistically in later relational encounters, with suspicion and mistrust, and anticipate being maltreated, hence more easily becoming tense and angry and use violent means in their relational discourse. Additionally, emotional and physical neglect could contribute to violence risk in severe mental illness by various routes, including lack of adult supervision and guidance in formative years and broad-based negative impacts on cognition and emotion (44, 45). The more extensive the history of adversities, the more the various negative psychological sequels of trauma might be at stage.

Consistent with the evidence of high trauma loads and presentation of trauma sequels in many forensic patients, implementing and further developing trauma informed care increasingly is part of quality improvement programs in forensic mental health services (21, 46–48). In these approaches, increased focus might be placed on assessing patients' life stories, and the difficulties they have faced (49). Trauma informed approaches may enhance staff's ability to understand the patients' traumatic past and how it can influence their (violent) behavior, minimize trauma triggers, de-escalate aggressive incidents, and contribute to less use of coercive measures. This may aid in co-creating individualized treatment and risk management plans, collaboratively involving the patient in his own recovery. Trauma informed care also encompasses addressing substance abuse, which stands in a dose-response relationship with childhood victimization (50) and constitutes an important risk factor for violent offenses in forensic patients, if necessary by joined-up working between forensic and addiction services (2, 51).

### Study Strengths and Limitations

Doing research in forensic mental health can be problematic by several reasons, including possible ethical barriers, patients being considered too ill or risky to study, lack of collaboration, and unwillingness to share information on violent behavior and vulnerable experiences such as past victimization. One consequence is selected participation and lack of representativeness of results from research (20). Being based on a quality register established to evaluate and inform treatment at a forensic ward, our study avoided questions of representativeness by including 100% of enrolled patients. At the same time, since our data were based on electronic patient files, they may underestimate the patients' extent of victimization during upbringing as well as their extent of violent acts in the community. At worst, we may have identified only the most severe instances of both victimization and violence. Moreover, the polyvictimization scale that we applied is likely to be a suboptimal measure of cumulative childhood victimization, since it did not include features such as degree of severity and duration of adversity types (52). In addition, we included only diagnosis as a measure of patients' mental health problems. Explanatory power likely would increase by including specific psychotic symptoms. Finally, the low number of participants limited our ability to explore more detailed associations in the data and be confident about the findings.

## Conclusions

In forensic mental health inpatients with diagnosis predominantly of paranoid schizophrenia and other schizophrenia spectrum and psychotic disorders, and where the majority had substance abuse, those with the most frequent acts of interpersonal violence in the community had been subjected to the most extensive maltreatments during upbringing. The findings add child adversities to established violent risk factors in forensic patients such as psychosis and substance abuse. They underline the need of trauma informed treatment approaches in forensic settings also for the most violent among the patients. Further studies with larger samples that apply cumulative measures of childhood victimization and violence may reveal more nuanced associations between these variables in forensic patients.

## Data Availability Statement

The datasets presented in this article are not readily available because the data are extracted from an internal quality assurance register at Vestre Viken. The Data Protection Office at Vestre Viken provided specific approval for this publication. This approval does not include sharing data from the register. Requests to access the datasets should be directed to the corresponding author.

## Ethics Statement

The studies involving human participants were reviewed and approved by the Vestre Viken hospital Data Protection Office for research at Vestre Viken, ref.no 16/00117-13, 16/00117-146, and 20/09213-1. Written informed consent for participation was not required for this study in accordance with the national legislation and the institutional requirements.

## Author Contributions

RF, GE, LS, and MK developed the study concept and study design. GE and LS performed the data collection. RF carried out data analysis. RF and SB interpreted the data. RF, GE, and LS drafted the paper. All authors provided critical revisions and approved the final version of the paper for submission.

## Conflict of Interest

The authors declare that the research was conducted in the absence of any commercial or financial relationships that could be construed as a potential conflict of interest.
